# Relative gene expression, micronuclei formation, and ultrastructure alterations induced by heavy metal contamination in *Pimelia latreillei* (Coleoptera: Tenebrionidae) in an urban-industrial area of Alexandria, Egypt

**DOI:** 10.1371/journal.pone.0253238

**Published:** 2021-06-23

**Authors:** Lamia M. El-Samad, Saeed El-Ashram, Dalia A. Kheirallah, Karolin K. Abdul-Aziz, Noura A. Toto, El Hassan M. Mokhamer

**Affiliations:** 1 Department of Zoology, Faculty of Science, Alexandria University, Alexandria, Egypt; 2 College of Life Science and Engineering, Foshan University, Foshan, Guangdong Province, China; 3 Faculty of Science, Kafrelsheikh University, Kafr El-Sheikh, Egypt; 4 Department of Zoology, Faculty of Science, Damanhour University, El Beheira, Damanhour, Egypt; Beni Suef University, Faculty of Veterinary Medicine, EGYPT

## Abstract

The present research aims to evaluate the impact of industrial processes and anthropogenic activities on the beetle *Pimelia latreillei* inhabiting the polluted site at Zawya Abd El- Qader, Alexandria, Egypt. Beetles were collected from the vicinity of five factories. The genotoxic effects of environmental exposures to industrial heavy metals were monitored using a broad range of assays, including energy-dispersive X ray microanalysis and X-ray diffraction (SEM and EDX)), qRT-PCR gene expression assay, micronuclei formation, and transmission electron microscope (TEM). Energy dispersive X-ray microanalysis for the soil and testicular tissues of beetles collected from the polluted site revealed a higher percentage of heavy metals than the beetles collected from the reference site (Sidi Kirier, Alexandria, Egypt). To analyze/monitor genotoxicity in *P*. *latreillei* sampled from the polluted site, the transcription levels of levels of heat shock proteins (Hsps) and accessory gland seminal fluid protein (AcPC01) in testicular tissues were recorded. The incidence of micronuclei (MN) formation in the testicular cells was also observed. Quantitative RT-PCR (RT-qPCR) analysis was carried out to detect the changes in the gene expression of the aforementioned proteins. Genes encoding heat shock proteins (*Hsp60*, *Hsp70*, and *Hsp90*) were significantly overexpressed (> 2-fold) in specimens sampled from the polluted site; however, *AcPC01* gene expression was under-expressed (<1.5-folds). The incidence of MN was significantly increased in specimens sampled from the polluted site. Ultrastructure anomalies (nuclear and cytoplasmic disruption) were also observed in the testicular cells of the beetles sampled from the polluted site compared to those sampled from the unpolluted site. Our results, therefore, advocate a need for adequate measures to reduce increasing environmental pollution in the urban-industrial areas.

## 1. Introduction

Heavy metals released from industrial processes and anthropogenic activities have an adverse effect on humans and the ecosystem [1, 2]. The World Health Organization (WHO) estimated that a million people died every year from diseases caused by pollution, most of them in developing countries [3, 4]. Inorganic pollutants released from industrial and agricultural sources contain heavy metals that result in soil pollution [5, 6]. The excess release of heavy metals into the soil makes them a major health concern worldwide [7, 8]. Most heavy metals have carcinogenic and/or mutagenic effects in addition to their cytotoxicity to healthy cells at low concentrations [9, 10].

As metal ions traverse cell barriers, the balance of extracellular and intercellular ions is interrupted, which affects membrane permeability [11]. Therefore, insects, including ground beetles have been used as bioindicators to monitor environmental pollution and in particular, soil pollution by heavy metals [12–15]. Ground beetles inhabit most of the biogeographical regions and are simply sampled from their habitats, and their bionomics and systematics are well studied [16]. Disturbance in the physiological mechanisms of the organisms is a reflection of environmental stress (Migula et al. 2004). Biochemical analysis is progressively used in ecotoxicological studies to monitor the ubiquity of xenobiotics [17]. Biochemical alterations have been attributed to the negative effects resulting from vulnerability to a contaminant [18]. A molecular biomarker is a significant tool for the evaluation of ecotoxicity in living organisms. Toxic compounds have a high affinity for electron pairs found in the amino acids [19]. Hence, elevation or inhibition in the activity of the enzymes is an indication of the damage caused by pollutants [20].

Genotoxic agents can induce several health disorders, such as structural abnormalities and growth retardation [21]. Consequently, there is a need for sensitive tests to monitor the genotoxicity of hazardous compounds found in the environment [22].

The physiological response of an organism exposed to a stressor triggers the synthesis of specific proteins to repair possible damage caused by such exposure. These proteins are named molecular chaperones or heat shock proteins (Hsps) [23, 24]. They have a role in protecting the stressed cells [25]. Hsp60, Hsp70, and Hsp90 are the highly conservative proteins and the most susceptible to stress factors in the organism’s cells (Cui et al. 2010; Sun et al. 2014). In insects, the exposure to stressors leads to a decrease in the rate of synthesis of most proteins, but Hsp expression increases [26, 27]. Several studies have implicated heat shock proteins (Hsps) in evaluating the toxic potential of different stressors in insects, particularly of heavy metals [26, 28].

In insects, seminal fluid proteins (SFPs) are produced by the accessory glands (AGs), vesicula seminalis, ejaculatory duct, ejaculatory bulb, and testes. The SFPs include protease inhibitors, lectins, prohormones, peptides, and protective proteins, such as anti-oxidants present in the ejaculate of all eukaryotes [29]. During mating, they are conveyed to the females, thereby inducing female post-mating responses. Changes in the levels of these proteins affect the reproductive success of both sexes [29, 30].

Micronuclei (MNs) are biomarkers used to monitor genotoxicity [31]. They are tiny cytoplasmic extrusion of chromatin that results from the breakage of the chromosomes during cell division or chromosomal delay in anaphase [32]. Very few studies have been conducted to evaluate genotoxic damage by different stressors using MN assay [33].

The ultrastructure of the internal organs of insects is a competent tool in determining the effect of toxins. The bioaccumulation of heavy metals into insects can be used as a monitor for environmental pollution. Insects possess special structures, spherites for accumulating trace metals [34]. Accumulation of heavy metals in insect organs influences cell viability and induces cellular damage, as well as cell apoptosis [14, 35–37]. Heavy metals may affect the regulation and control mechanisms of the reproductive process, leading to spermatogenetic alterations, which, in turn, can result in the production of damaged spermatozoa [38–40]. Ultrastructure anomalies in insects’ testes induced by heavy metal pollution have been reported in a few studies [14, 35, 37].

Using a biomonitoring beetle, *Pimelia latreillei*, this study clarified the genotoxic effect of heavy metals originating from anthropogenic sources and industrial effluents. We also observed the ultrastructure damages to the testicular cells, which may be caused by heavy-metal pollution.

## 2. Materials and methods

### 2.1. Ethics statement

The ethical rules for animal regulations were followed and approved by Faculty of Science, Alexandria University committee in March 2018 (Alex-01-2018). All animal procedures were conducted in accordance with the local Guiding Principles for the Care and Use of Laboratory Animals as adopted and promulgated by Alexandria University.

### 2.2. Study sites

Two sites were chosen for sampling the coleopteran insects. The sample locations were in public areas. Site A at Sidi Kirier, north coast of Alexandria, Egypt (latitude 31.016250°N and longitude 29.635663°E), was considered the reference site. Some ornamental plants, grasses, and shrubs were cultivated at this site. Site B at Zawya Abd El-Qader, southwest Alexandria, Egypt (latitudes 30° 33’ - 31° 30’ N and longitudes 29° 50’ - 30° 45’ E), was considered as the polluted site. This area covered a vast cultivated land representing most Abis and El-Nahada farms. These lands were subjected to aerosol deposition from various industrial activities located in the western part of Alexandria city [Site B1, Egyptian Petrochemicals Company or EPC (latitude 31.009206°N and 29.848589°E; Site B2, Alexandria Carbon Black (latitude 30.995080°N and longitude 29.848739°E); Site B3, Pirelli Tires Company (latitude 30.997497°N and longitude 29.846674°E); Site B4, Sidi Krier Petrochemical Company or Sidpec (latitude 31.004389°N and longitude 29.839531°E); and Site 5, Egyptian Ethylene Company or Ethydco (latitude 31.011130°N and longitude 29.832288°E)]. From meteorological data, the wind direction was found to be northwest (average speed between 2.75 m/s and 7.14 m/s), which might accelerate the delivery of contaminants over a long distance.

### 2.3. Sampling procedure

Live specimens of *P*. *latreillei* were collected randomly from ten sampling areas (1 m^2^ each) at each site in June 2018. Simultaneously with the beetle collection, soil samples were collected at a depth of 25 cm below the surface from the mentioned sites. The ten areas at Zawya Abd El Qader (the polluted site) were selected near the aforementioned companies (two areas around each company). The mean air temperature in June ranged from 28°C to 36°C and the mean relative humidity was 65%, with nearly no differences between the two sites. About 200 insects were collected from each site. The specimens were sexed, and about 90 males were preserved alive in local soil and plants in glass containers until processing. Beetles were anaesthetized with absolute ethanol (95%), then dissected under a dissecting microscope in a drop of Ringer’s physiological solution. The abdominal cavity was opened and the testes were extracted.

### 2.4. Studied insect

The specimens were identified at the Faculty of Agriculture, Alexandria University (Department of Entomology) as *Pimelia latreillei*. The studied insect belonged to Tenebrionid beetles.

### 2.5. Determination of heavy metals in the soil and testicular tissues of *P*. *latreillei*

Energy-dispersive X-ray microanalysis (EDX) was used to determine the percentages of different metals in the sieved soil and testicular tissues. This analysis was applied using a JEOL (JSM-5300) scanning microscope at the Electron Microscope Unit (E.M.), Faculty of Science, Alexandria University, Egypt. The accuracy of the analytical results was determined using eight samples of soil from each site and testicular tissues obtained from eight male beetles.

The identity of each peak was assigned automatically by the SEM EDX software. Line intensity was measured for each element in the sample and for the same elements in calibration standards of known composition. At X500, a stationary spot was analyzed at random for 110 s.

### 2.6. mRNA expression of heat shock proteins (Hsps) and seminal fluid (AcPC01) encoding genes

#### 2.6.1. Isolation of total RNA

Total RNA was isolated from eight samples of testicular tissues and accessory glands of male *P*. *latreillei* with TRIzol® Reagent (Invitrogen, Germany). To ensure DNA digestion, 1 U of RQ1 RNAse-free DNAse (Invitrogen, Germany) was added to the RNA, and the mixture was re-suspended in DEPC-treated water. Total RNA purity was assessed by the 260/280 nm ratio (between 1.8 and 2.1). To ensure integrity, ethidium bromide stain analysis of 28S and 18S bands by formaldehyde-containing agarose gel electrophoresis was performed. Aliquots were used for reverse transcription (RT).

#### 2.6.2. Reverse transcription (RT) reaction

Poly (A) + RNA isolated from testicular tissues and accessory glands of *P*. *latreillei* was reverse transcribed into cDNA in a total volume of 20 μl using Revert AidTM First Strand cDNA Synthesis Kit (MBI Fermentas, Germany). From the total RNA, 5 μg was used with a master mix (MM) consisting of 50 mM MgCl2, 5x reverse transcription (RT) buffer (50 mM KCl; 10 mM Tris-HCl; pH 8.3), 10 mM of dNTP, 50 μM oligo-dT primer, 20 U ribonuclease inhibitor (50 kDa recombinant enzyme to inhibit RNase activity), and 50 UM- MuLV reverse transcriptase. Each sample mixture was centrifuged for 30 s at 1000 g. The mixture was then transferred to the thermo-cycler (Biometra GmbH, Göttingen, Germany). The RT reaction started at 25°C for 10 min, continued at 42°C for 1 h, and was stopped after heating at 99°C for 5 min, followed by cooling in an ice chamber.

#### 2.6.3. Real Time-Polymerase chain reaction (RT-qPCR)

Step One™ Real-Time PCR System from Biosystems (Thermo Fisher Scientific, Waltham, MA, USA) was used to determine the beetles’ cDNA copy number. PCR reactions were set up in 25 ml reaction mixtures containing 12.5 ml 1× SYBR® Premix Ex TaqTM (TaKaRa, Biotech. Co. Ltd.), 0.5 ml 0.2 mM sense primer, 0.5 ml 0.2 mM antisense primer, 6.5 ml distilled water, and 5 ml of cDNA template.

**Fig 1:**
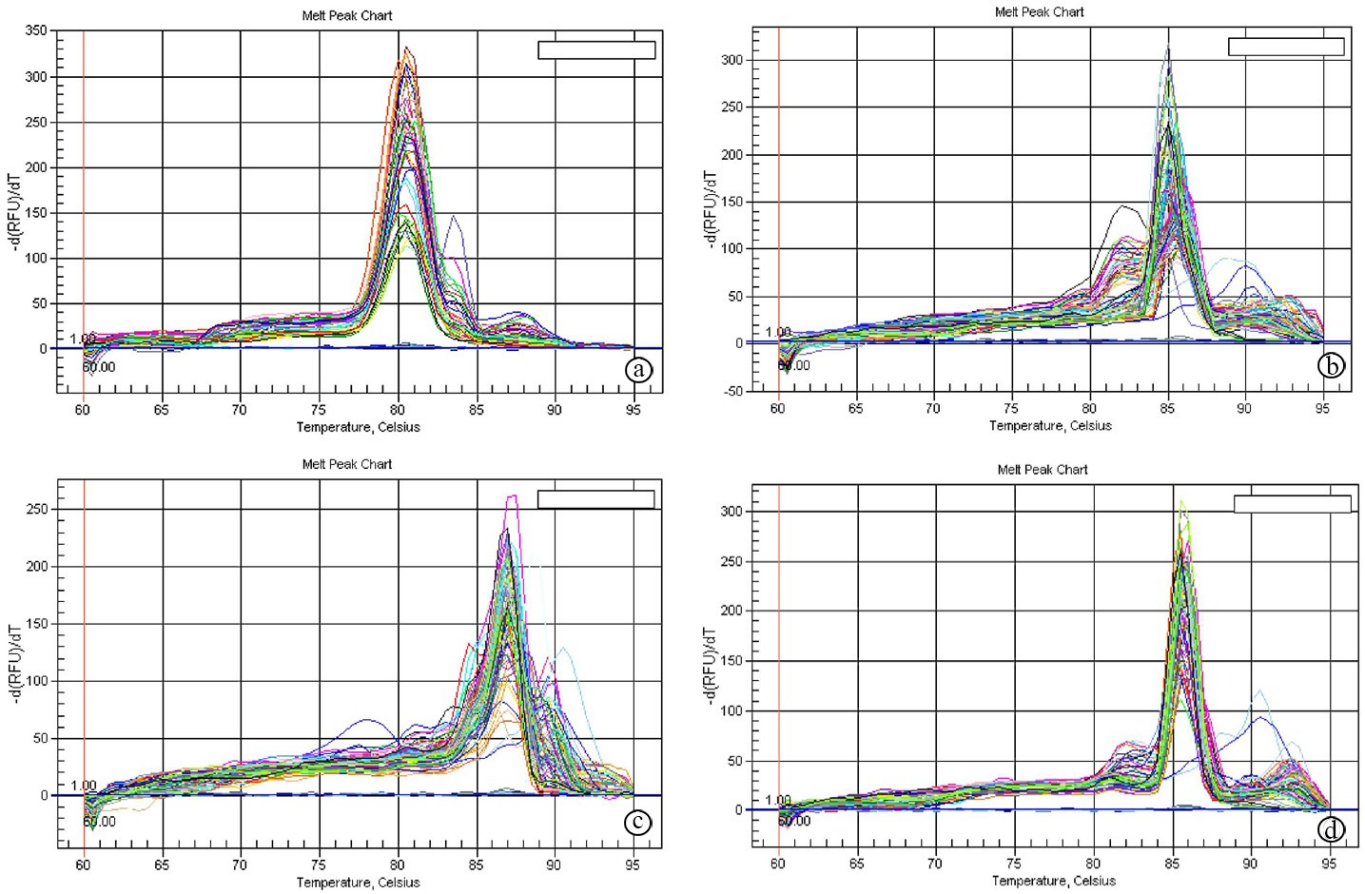
Melting curves of *Hsps* and *AcpC01* genes. **a:** Melting curve of *Hsp60* gene, **b:** Melting curve of *Hsp70*, **c:** Melting curve of *HspP90* gene, **d:** Melting curve of *AcpC01* gene.

The reaction program was divided into 3 steps. Step (1) was at 95.0°C for 3 min. Step (2) consisted of 40 cycles in which each cycle was subdivided into 3 steps: (a) at 95.0°C for 15 s; (b) at 55.0°C for 30 s; and (c) at 72.0°C for 30 s. Step (3) consisted of 71 cycles which started at 60.0°C and then increased about 0.5°C every 10 s up to 95.0°C. Primer quality was measured using melting curve analysis that was executed at the end of each RT-qPCR (Fig 1). Each experiment included a distilled water negative control. The sequences of the specific primer of the genes used in accordance with Liu et al. (2014) [41], Rodríguez-García et al. (2015) [42], and Cai et al. (2017) [43], are listed in Table 1. The relative quantification of the target to the reference was determined using the 2^−ΔΔCT^ methods.

**Table 1:** Primers sequence used for RT-qPCR.

Gene name	Primer sequense (5′-3′)	GenBank accession No., Full-length cDNA library, & Amplicon size	References
*Hsp60*	F: GCT GTA TGT CCG CCG TGT A	Genbank acc. n.: KU323593 Amplicon size: 427 bp	Cai et al. (2017) [43]
	R: GGG AGT CTT CGT GAA TGC C		
*Hsp70*	F: TGG CGG CAA ACC GAA GAT	Genbank acc. n.: KU159184 Amplicon size: 576 bp	
	R: CGC TGG CAC CGT AAT GAC		
*Hsp90*	F: GAG GAA GGT ATT GTA GCA GG	Genbank acc. n.: KU159185 Amplicon size: 313 bp	
	R: AGC GGT CGT CAA GAG GGA TG		
AcPC01	F: GTA TTC CAT TGT GTC CAC CAC CTC CGG	Genbank acc. n.: KP164546.1 Amplicon size:128bp	Liu et al. (2014) [41]
	R: TGG TGG ACA AGG TGG ACA ACA TGG AAC		
*β-actin*	F: CTC TGC TAT GTA GCC CTT GAC TT	Genbank acc. n.: KU884974.1 Amplicon size: 156 bp	Rodríguez-García et al. (2015) [42]
	R: GCA GTT GTA GGT GGT TTC GTG		

*Hsp60*: heat shock protein 60 encoding gene, *Hsp70*: heat shock protein 70 encoding gene, *Hsp90*: heat shock protein 90 encoding gene, *AcPC01*: accessory gland seminal fluid protein encoding gene, *β-actin*: Beta actin encoding gene.

### 2.7. Micronucleus (MN) test

Samples of beetle testes were prepared for MN analysis. The testes were immersed in saline solution (128.3 mM NaCl, 16.7 mM Na2HPO4, 19.9 mM KH2PO4), incubated in tap water as a hypotonic treatment for 50 min to let the cells swell, allowing the mononuclear and binuclear to separate. 3 μg/ml of cytochalasin B was used to block cytokinesis. Testis was spread on coded slides, fixed in Water: Ethanol: Acetic Acid by vol (4:3:3) for 20 min, Ethanol: Acetic Acid,1:1(v/v) for 30 min, and Acetic Acid (100%) for 24 h, air dried, stained with Giemsa dye 1M diluted in 30 M buffer (0.06M sodium citrate buffer, pH: 6.8) for 10 min. About 1000 testicular cells were scored for each slide under a light microscope at a magnification of 1000× to determine the frequency of MN [33, 44]. Other nuclear abnormalities were also noticed in the cells, including nuclear buds, karyorrhexis, karyolysis, binucleated cells, and heterochromatin [45, 46].

### 2.7.1. Micronuclei identification

**Fig 2:**
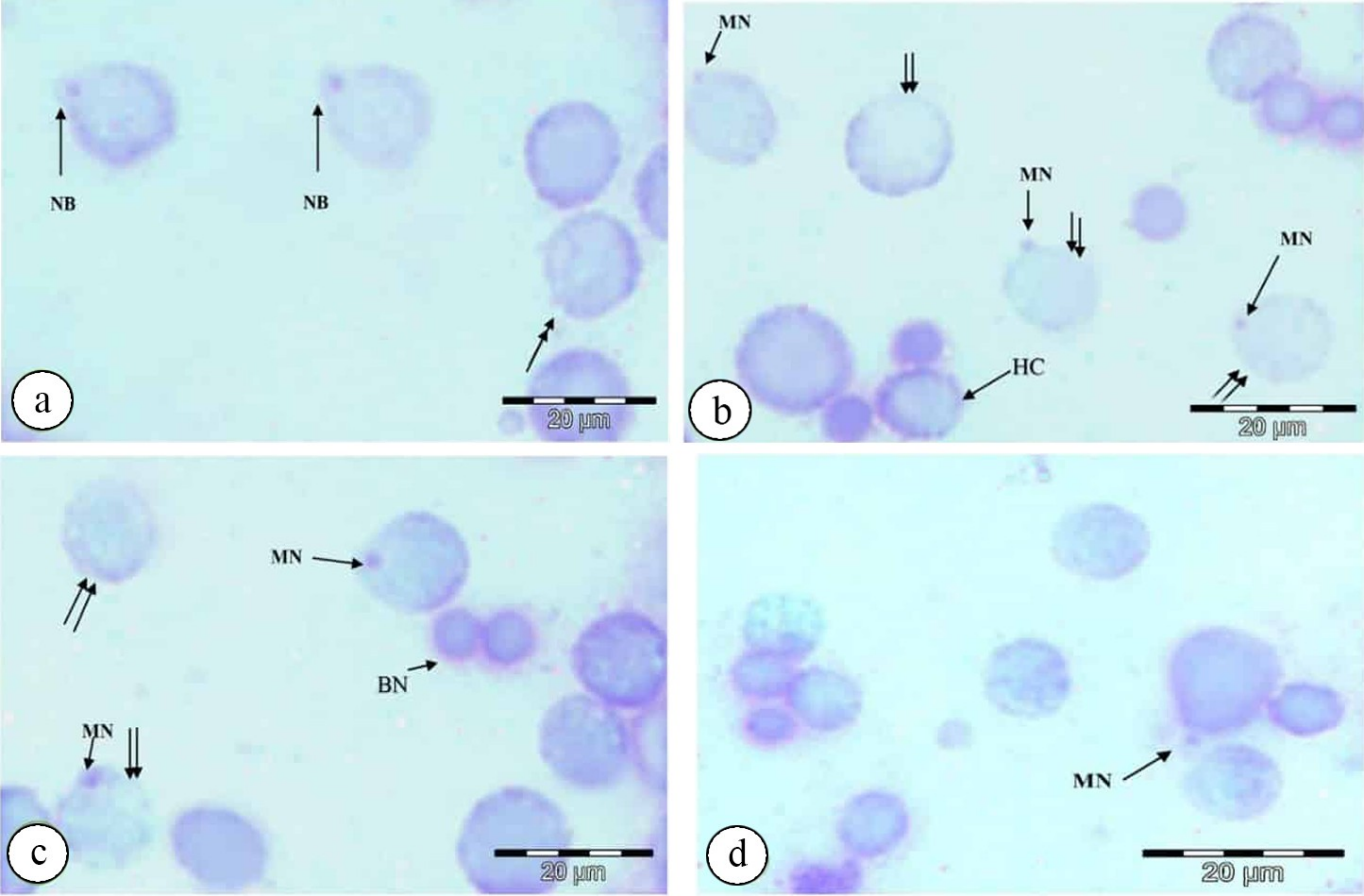
Photomicrographs of nuclear abnormalities in the testicular cells of *P*. *latreillei* collected from the polluted site, stained with Giemsa. **a:** nuclear bud (NB) and karyorrhexis (double head arrow), **b:** micronucleus (MN), karyolysis (double arrow), and heterochromatin (HC), **c:** binucleated cell (BN), micronucleus (MN), and karyolysis (double arrow), **d:** micronucleus (MN).

Micronuclei (MN) are illustrated in Fig 2 according to the following criteria: (1) the structure and staining of MN must be similar to the main nuclei; (2) MN are not connected to the main nuclei, but they may touch the main nuclei; (3) MN should have spherical structures; (4) MN diameter should not be greater than 1/3 core diameter.

### 2.8. Preparation of testes for ultrastructure analysis

Testes were fixed immediately in 4% formaldehyde and 1% glutaraldehyde (_4_F_1_G) in 0.1 M phosphate buffer solution (pH 7.2) at 4°C for 3 h, followed by post-fixation with 2% osmium tetroxide (OsO_4_) in the same buffer for 2 h. A buffer was used to wash the samples, which were dehydrated at 4°C through a graded series of ethanol, then embedded in Epon-Araldite mixture in labeled beam capsules. Ultrathin sections (0.06–0.07 μm thick) were cut from the testes for examination under a transmission electron microscope (TEM). The ultra-thin sections were placed on 200 mesh copper grids, which were double-stained with uranyl acetate for 30 min and lead citrate for 20–30 min (Reynolds 1963). Electron micrographs were taken at several magnifications. Scoping and photographing the grids were achieved by JEOL 100 CX TEM, at Electron Microscope Unit, Faculty of Science, Alexandria University, Egypt.

### 2.9. Data analysis

Data analysis was performed using the IBM SPSS software package version 20.0 (Armonk, NY: IBM Corp) [47]. The Shapiro‒Wilk test was used to verify the normality of the distribution of variables. Data were analyzed by a Student’s t-test (Sokal and Rohlf 1981) to determine the difference between the two studied sites for normally distributed quantitative variables. The significance of the obtained results was judged at *p* ≤ 0.05.

## 3. Results

### 3.1. X-ray analysis of soil samples and testicular tissues of *P*. *latreillei* collected from the inspected sites

Trace metal percentages were obtained from the X-ray analysis of sieved soil and the testicular tissues of *P*. *latreillei* sampled from the inspected sites (Tables 2 and 3).

**Table 2:** Trace metal percentages (%) in sieved soil samples from reference and polluted sites (site A & B), n = 8.

Metal Site	Site A	Site B	*P*
**Mg**	0.2 ± 0.07	1* ± 0.1	<0.001
**Ca**	5.4 ± 0.2	3.5* ± 0.3	0.006
**K**	1.7 ± 0.3	2.6 ± 0.3	0.09
**Na**	1.5 ± 0.6	16.9* ± 1.5	<0.001
**Zn**	0.3 ± 0.08	1.1* ± 0.07	<0.001
**Cu**	0.5 ± 0.1	2.4*± 0.4	<0.001
**Fe**	3 ± 0.4	8.2* ± 0.5	<0.001
**Al**	0.6 ± 0.2	4.1*± 1.3	0.022
**Pb**	ND	1.4*± 0.06	<0.001
**Cd**	ND	14*± 0.1	<0.001
**Ti**	0.3± 0.1	1.9*± 0.2	<0.001
**Si**	21.7± 0.9	79*±3.1	<0.001

For each metal, the percentage expressed by using minimum–maximum values and mean (n = 8) using Student t-test

*: Statistically significant at (*p* ≤ 0.05), ND: Not detected.

**Table 3:** Trace metal percentages (%) in testicular tissues of *P*. *latreillei* collected from the reference and polluted sites (site A & B), n = 8.

Metal Site	Site A	Site B	*p*
**Ca**	3.5 ± 0.06	5.7* ± 0.4	0.01
**K**	7.3 ± 0.2	ND	0.000
**Na**	8.4 ± 0.2	13.7* ± 1.7	0.05
**Zn**	4.2 ± 0.04	6.4* ± 0.5	0.02
**Cu**	3.6 ± 0.2	18* ± 4.7	0.05
**Fe**	ND	1.6* ± 0.1	0.002
**Al**	5.4 ± 0.1	21* ± 4	0.03
**Pb**	ND	3.4* ± 0.1	0.000
**Cd**	ND	1.1* ±0.1	0.006

For each metal, the percentage expressed by using minimum–maximum values and mean (n = 8) using Student t-test

*: Statistically significant at (*p* ≤ 0.05), ND: Not detected.

Twelve elements, Mg, Ca, K, Na, Zn, Cu, Fe, Al, Pb, Cd, Ti, and Si, were detected in the soil from site B and ten elements from site A (Pb and Cd were absent). A significant elevation in the percentages of metals was reported at the site B compared with those at site A, except for K, with detection of Pb and Cd (Table 2). However, only six elements were present in the testicular tissues of *P*. *latreillei* sampled from site A (Ca, K, Na, Zn, Cu, and Al) and eight elements in the samples from site B (Ca, Na, Zn, Cu, Fe, Al, Pb, and Cd). A significant elevation in the percentages of Ca, Na, Zn, Cu, and Al was observed in the testicular tissue of beetles collected from the site B compared with those at site A, except for K (not detected), with detection of Fe, Pb, and Cd (Table 3).

### 3.2. Gene expression of Heat shock proteins (Hsp60_,_ Hsp70_,_ Hsp90) and seminal fluid protein (AcPC01) in testicular tissues and accessory glands of *P*. *latreillei* collected from the inspected sites

**Fig 3:**
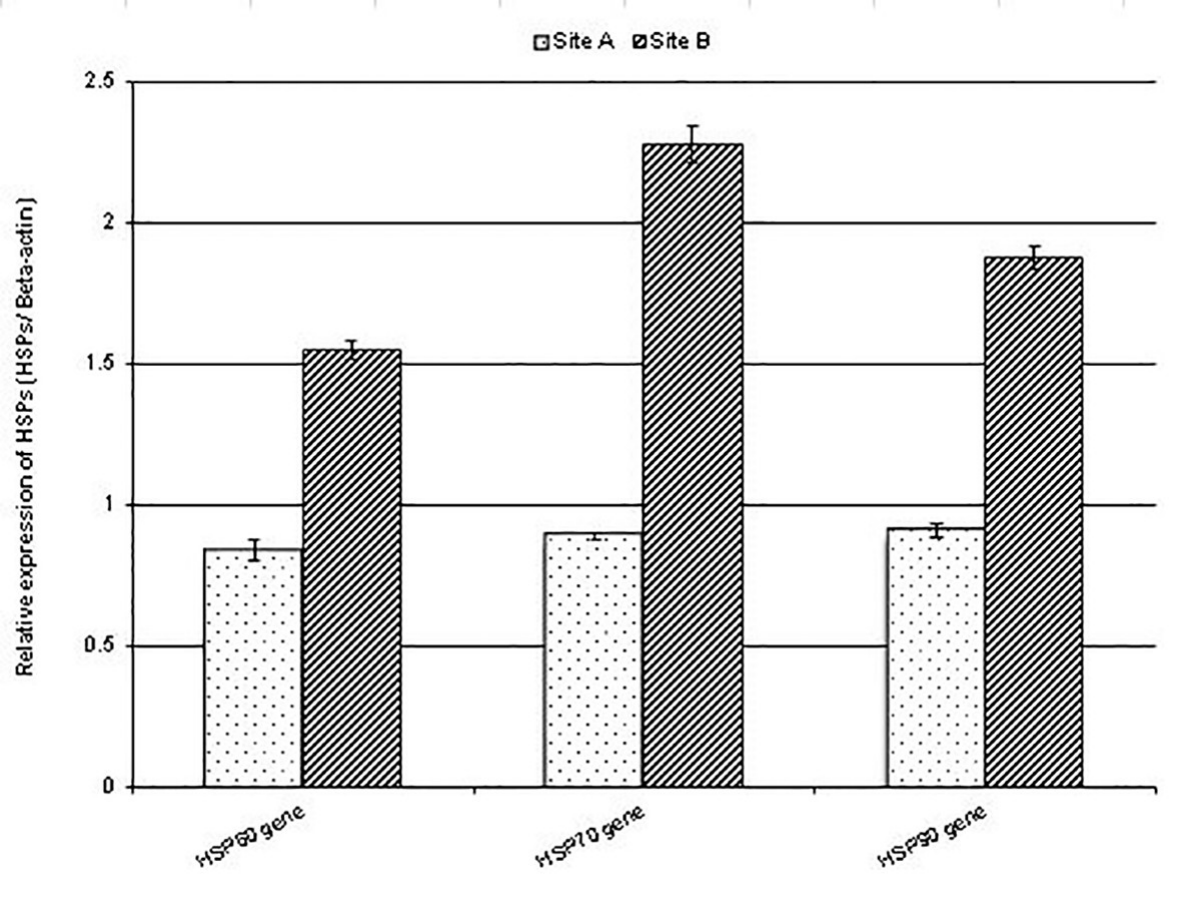
Expression levels of heat shock protein-encoding genes (*Hsp60*, *Hsp70*, and *Hsp90*) in testicular tissues of male beetles collected from the reference and polluted sites. Data are represented as mean ± SE, *p* <0.05.

**Fig 4:**
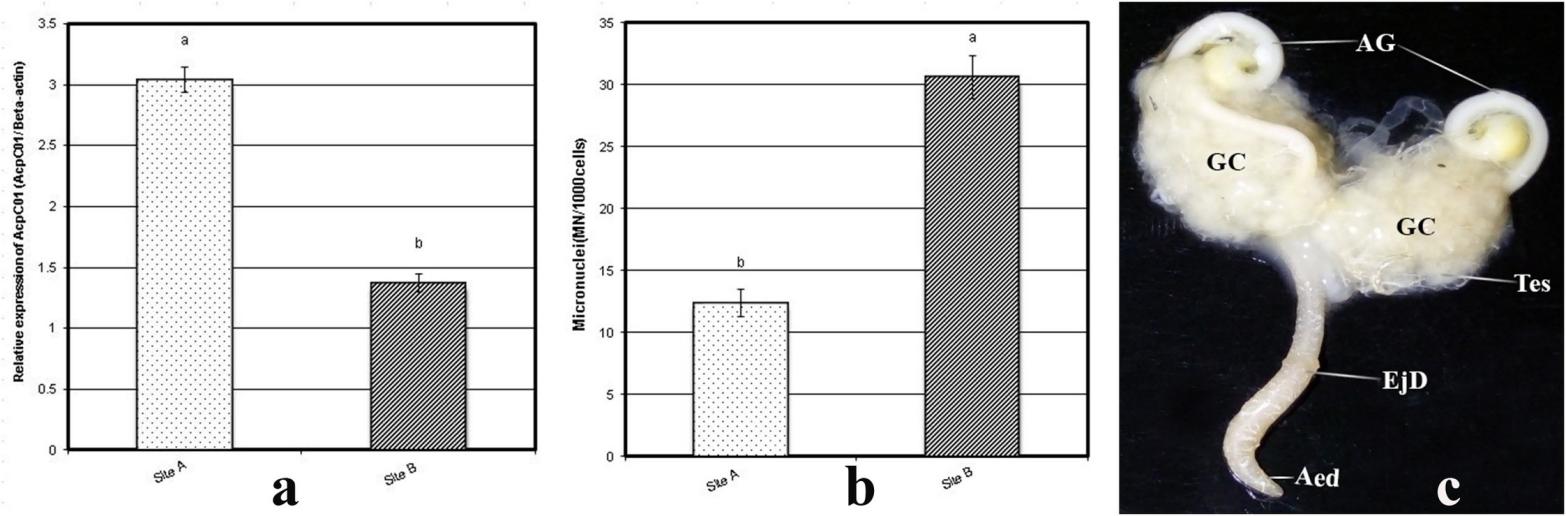
Expression levels of the seminal fluid encoding gene (*AcPC01*) in the accessory glands of male beetles collected from the reference and polluted sites. Data are represented as mean ± SE, p<0.05. **a&b:** Frequency of micronuclus formation (MN) in the testicular cells of *P*. *latreillei* collected from reference and polluted sites. Data are represented as mean ± SE, p<0.05. c: Photograph of the male reproductive system of *P*. *latreillei*. Testis (Tes), germinal cyst (GC), accessory gland (AG), ejaculatory duct (EjD), aedeagus (Aed).

cDNA obtained from testicular tissues was used as the template for RT-qPCR, which was conducted to investigate the gene expression patterns of Hsp60, Hsp70, and Hsp90 in the testicular tissues of *P*. *latreillei*. The *Hsp60* (GenBank Accession: KU323593, full-length cDNA library: 2143 bp, amplicon size: 427bp), *Hsp70* (GenBank Accession: KU159184, full-length cDNA library: 1947 bp, amplicon size: 576bp), and *Hsp90* (GenBank Accession: KU159185, full-length cDNA library: 2385 bp, amplicon size:313bp) transcripts were detected to be highly significant, being more than 2-fold in the testicular tissues of beetles collected from the polluted site in comparison with the expression observed in the testicular tissues of beetles collected from the reference site. In particular, relatively high mRNA expression levels of *Hsp70* were observed in samples from the polluted site (Fig 3). However, a significant inhibition in *AcPC01* (GenBank Accession: KP164546.1, full-length cDNA library: 20 bp, amplicon size: 5128 bp) transcript level, being less than 1.5-fold, was observed in the accessory glands of male *P*. *latreillei* collected from the polluted site, compared with that of the reference site (Fig 4A).

### 3.3. Micronucleus assay

The incidence of micronuclei in the testicular cells of *P*. *latreillei* due to the effect of heavy metals is presented in Fig 4B. Data are expressed as Mean ± SE of five replicates. Micronuclei frequency were expressed in 1000 analyzed testicular cells. The number of micro-nucleated cells among the polluted group was highly significant, being 30.6±1.72 compared with the micro-nucleated cell number in the control group (12.4±1.08).

### 3.4. Macroscopic observations

The male reproductive organs of *P*. *latreillei* consist of two testes, which are bulblike structures encased in a peritoneal sheath and composed of follicles, the calyx, the vas deferens, and the vesicula seminalis at each side combined into the ejaculatory duct and leading to the aedeagus. The ejaculate received two accessory glands. There were no external anatomical abnormalities recognized in the testes of beetles collected from the polluted site, compared to the reference group (Fig 4C).

### 3.5. Ultrastructure observations of the testis of *P*. *latreillei* collected from the inspected sites

**Fig 5:**
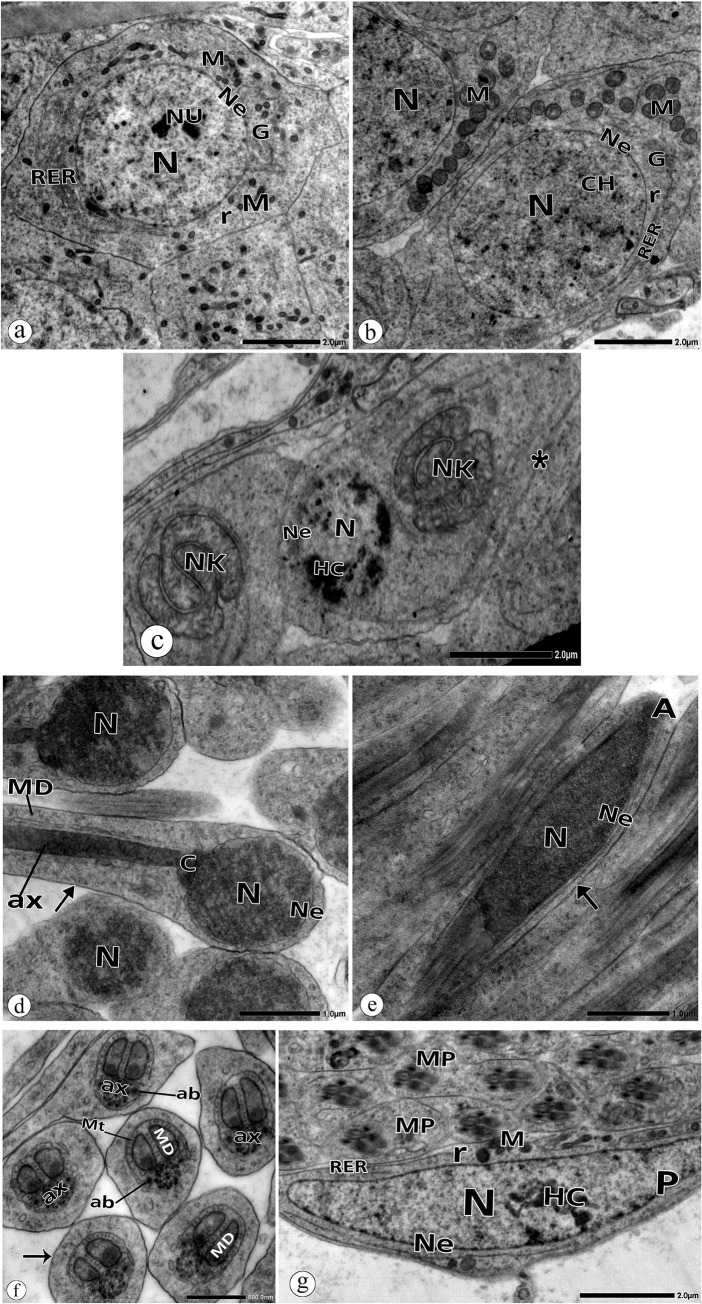
Electron micrographs of spermatogenic cell in the testis of *P*. *latreillei* collected from the site A. **a:** Spermatogonia with nucleus (N), regular nuclear envelope (Ne), mitochondria (M), rough endoplasmic reticulum (RER), Golgi complex (G), free ribosomes (r). **b:** Spermatocyte with euchromatic nucleus (N), regular nuclear envelope (Ne), mitochondria (M), rough endoplasmic reticulum (RER), Golgi Complex (G), and free ribosomes(r). **c:** Early spermatids with heterochromatic (HC) nucleus (N), nuclear envelope (Ne), nebenkern (NK), connecting bridge (*). **d:** Late spermatid with nucleus (N), nuclear envelope (Ne), centriole (C), axoneme (ax), plasma membrane (arrow). **e**: sperm with acrosome (A), nucleus (N), nuclear envelope (Ne), plasma membrane (arrow). **f:** Middle pieces of early spermatids with axoneme (ax), MD: mitochondrial derivatives (MD), microtubules (Mt), accessory body (ab), plasma membrane (arrow). **g:** Parietal cell (P) with heterochromatic (HC) nucleus (N), regular nuclear envelope (Ne), mitochondria (M), rough endoplasmic reticulum (RER), free ribosomes.

The present results are the first describing the testicular structure of the studied beetle, *P*. *latreillei*. There are no previous studies that describe such a structure. Electron micrographs of the testis of *P*. *latreillei* sampled from the reference site (site A) showed euchromatic spermatogonia with a large spherical nucleus, one or two dense nucleoli, and a regular nuclear envelope. Their cytoplasm contained mitochondria distributed around the nucleus. Rough endoplasmic reticulum, Golgi complex, and free ribosomes were also observed (Fig 5A). The spermatocytes appeared with a larger nucleus and homogenous chromatin. The mitochondria redistributed on one side of the nucleus, preparing for nebenkern formation in the initial spermatids (Fig 5B). The initial spermatids were diagnosed by their small round nuclei with condensed chromatin and round nebenkern formed by the fusion of the mitochondria (Fig 5C). The interconnected bridges between the initial spermatids were noticed, as they arose from a single spermatocyte (Fig 5C).

Late spermatids had an oval nucleus, a centriole, and an axoneme. The nebenkern was divided into two mitochondrial derivatives, which extended posteriorly around the axoneme (Fig 5D), while spermatozoa had a more dimensional nucleus, a conical acrosome, and flagellum (Fig 5E). Cross-sections through the flagellum showed nine accessory tubules and nine doublet tubules surrounding two central tubules. Hence, the axoneme was seen as having a 9+9+2 tubular pattern (Fig 5F). Two accessory bodies were also noticed (Fig 5F).

The electron micrographs marked out parietal cells that form the germinal cyst borders. They are characterized by their large polymorphous nucleus with a few patches of chromatin near the nuclear envelope. Their cytoplasm contained mitochondria, free ribosomes, and rough endoplasmic reticulum (Fig 5G).

**Fig 6:**
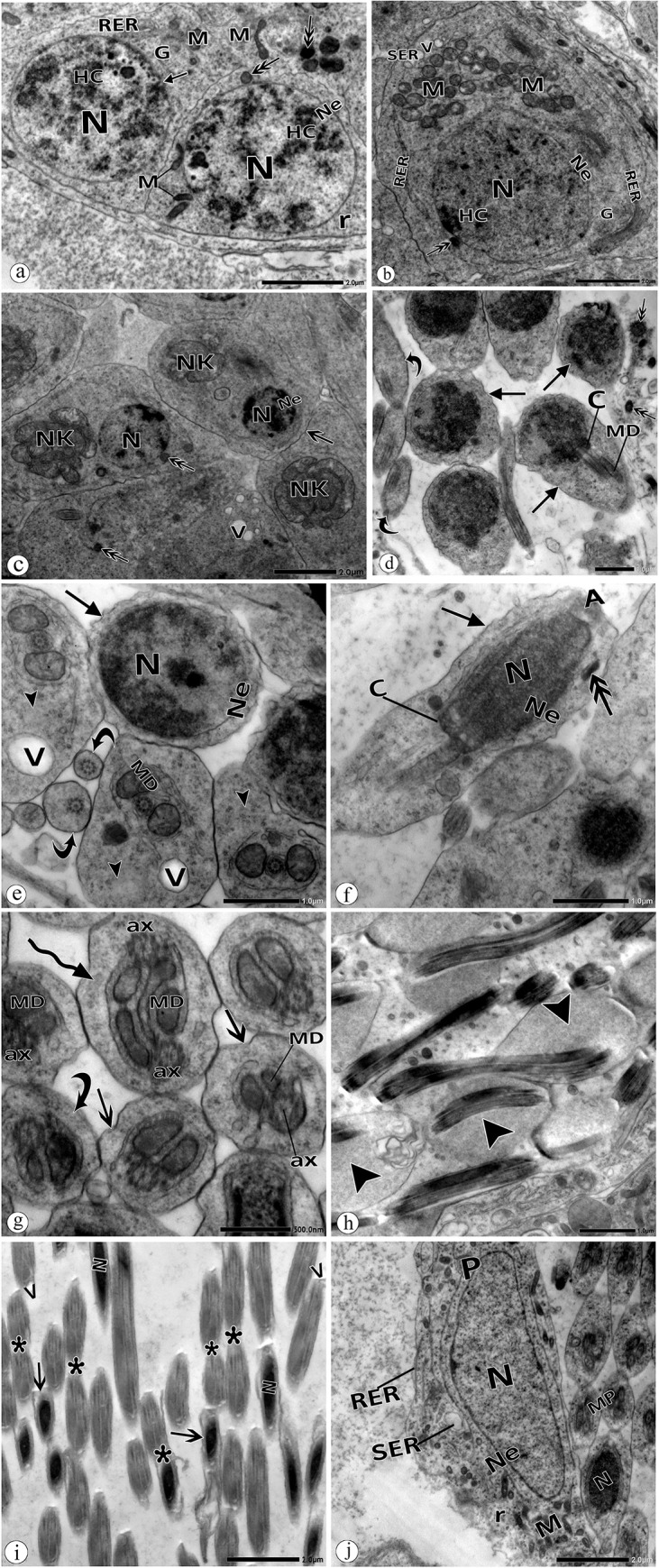
Electron micrographs of spermatogenic cell in the testis of *P*. *latreillei* collected from the site B. **a:** Spermatogonia (Sg) with nucleus (N), nuclear envelope (Ne), globular inclusion body (arrow), heterochromatin (HC), dense mitochondria (M), dense vesicle (double head arrow), free ribosomes (r). **b:** Spermatocyte with nucleus (N), nuclear envelope (Ne), mitochondria (M), dilated rough (RER), and smooth (SER) endoplasmic reticulum, free ribosomes (r), vacuoles (V), dense vesicle (double head arrow). **c:** Early spermatid with abnormal chromatin condensation, disintegrated nebenkern (NK), convoluted plasma membrane (arrow), vacuolated cytoplasm (V), dense vesicle (double head arrow), nucleus (N), nuclear envelope (Ne). **d:** Abnormal head morphology of late spermatids with convoluted plasma membrane (arrow) and malformed middle pieces (curved arrow), centriole (C), mitochondrial derivatives (MD), d: dense vesicle (double head arrow). **e:** Spermatid with irregular nuclear envelope (Ne), convoluted plasma membrane (arrow), abnormal middle pieces with residual cytoplasm (head arrow), disintegrated mitochondrial derivatives (MD), middle pieces lacking mitochondrial derivatives (curved arrow), vacuoles(V). **f:** Spermatids with irregular nuclear envelope (Ne), convoluted plasma membrane (arrow), centriole (C), nucleus (N), acrosome (A), dense vesicle (double head arrow). **g:** Middle pieces with degenerated axoneme (ax), degenerated mitochondrial derivatives (MD), convoluted plasma membrane (double head arrow). Note: spermatid with a double tail (curved arrow). **h:** Spermatids with residual cytoplasm (head arrow). **i:** Spermatozoa with the convoluted plasma membrane (arrow). Note: agglutinated sperms, head to tail, and tail to tail (*). N: nucleus, v: vacuoles. **j:** Hypertrophied parietal cell (P) with high phagocytic activity. N: nucleus, Ne: nuclear envelope, M: mitochondria, RER: rough endoplasmic reticulum, SER: dilated smooth endoplasmic reticulum, r: free ribosomes.

The electron micrographs of the testicular cells of the polluted group showed some anatomical anomalies. In spermatogonia, there were some nuclear deformations, such as indentation of the nuclear envelope and formation of globular inclusion bodies. Dense vesicles were noted in the cytoplasm (Fig 6A). The degenerative changes in the spermatocyte appeared more pronounced in the cytoplasm. These changes included lysis of mitochondrial matrices, the appearance of dense vesicles, and vacuolated cytoplasm. In the nucleus, some discrete patches of heterochromatin were observed (Fig 6B).

Early spermatids appeared with abnormal chromatin clumping (Fig 6C). Cytoplasmic deformities included vacuolations, nebenkern disintegration, dense vesicles, and convolution of the plasma membranes (Fig 6C). Many anomalies were noticeable in late spermatids, such as abnormal head morphology with aberrant chromatin and irregular nuclear envelope (Fig 6D–6F). Transverse sections through their flagella showed disintegrated mitochondrial derivatives, degenerated axonemes, vacuolated and residual cytoplasm, and convolution of plasma membranes (Fig 6E, 6G and 6H). Agglutinated spermatids (tail to tail), and spermatids with a double tail were also noticed (Fig 6G).

Variable deformities were also detected in the spermatozoa. Sperms failed to discard their residual cytoplasm (Fig 6H). Convolution of the plasma membrane and agglutinated sperms (head to tail and tail to tail) were frequently observed (Fig 6I).

The parietal cells were seen to be hypertrophied, with distended cytoplasm and vigorous phagocytic activity in malformed spermatozoa (Fig 6J). A dilated smooth endoplasmic reticulum was observed in the cytoplasm (Fig 6J).

## 4. Discussion

Employing insects in biomonitoring program is a functional ecological indication [12, 14, 15, 35, 37, 48, 49]. In the present study, the urban site is prone to industrial heavy metal pollution that might be released from the local factories. Therefore, agricultural soils are highly polluted with various heavy metals resulting from anthropogenic activities and industrial processes.

In this study, we used x-ray microprobe analysis to detect heavy-metal concentrations in the soil and insect testicular tissues. There was a significant elevation in heavy-metal percentages at the polluted site, particularly Cu, Zn, Al, Cd, and Pb compared with the control site. X-ray analysis is an effective tool for detecting trace metal in biological specimens [15, 50]. Our results align with previous studies that reported the toxic effect of heavy metals on aquatic and terrestrial insects collected from industrial areas [14, 37, 49–52]. Azam et al. (2015) [51] stated that the elevation in heavy metals percentages in animal bodies correlates site pollution.

RT-qPCR primers were used to amplify homologous sequences in the available coleopteran species [42, 43]. The three tested heat shock proteins (Hsps) showed highly significant transcript levels in response to heavy-metal pollution at site B compared with the reference site (site A). It was stated earlier by Qin et al. (2003) [53] that between 1.5 to 4-fold increase in the transcriptional activities of these molecular chaperones was found to be a significant induction. Dou et al. (2017) [54] and Cheng et al. (2018) [55] reported that *Hsp60*, *Hsp70*, and *Hsp90* transcripts were expressed throughout insect development, suggesting a development regulatory role. Elevation in *Hsp* mRNA levels in insects due to heavy metal pollution was reported by Shu et al. (2011) [56] and Zhao et al. (2010) [57]. Induction of Hsp60, Hsp70, and Hsp90 protects against environmental stresses [41, 58, 59], although in our study *Hsp* mRNA levels were upregulated in the tested insect sampled from the polluted site, particularly Hsp70 gene. Hsp70 protein is the most dominant protein found in the early instars of insects and helps them to overcome adverse conditions [60]. Hsp70 protein may guard cells against metal-induced chromosome aberrations through different mechanisms that facilitate cell cycle regulation and reduce genomic instability [61]. It also stops the aggregation of the broken down proteins, leading to many serious injuries in the stressed cells [60]. Our results are in agreement with Doğanlar et al. (2014) [62], who exposed adult *Drosophila melanogaster* to different concentrations of metal mixture (Fe, Cu, Cd, and Pb). They reported that the expression of *Hsp* genes was altered by increasing the exposure time and that Hsp70 was the more expressed gene. Moreover, Braeckman et al. (1997a), Braeckman et al. (1997b), and Kafel et al. (2012) [63–65] observed an increase in the expression level of Hsp70 in *Aedes albopictus* and *Spodoptera exigua* exposed to cadmium. Joshi and Tiwari (2000) [66] noticed that environmental chemical pollutants, such as arsenate and mercury cause the induction of a common set of gene loci encoding heat shock proteins in the Australian sheep blowfly, *Lucilia cuprina*.

SFP analysis gives the perception of evolutionary patterns of reproductive traits [30]. Understanding reproductive molecules and their mechanisms provide opportunities to isolate species [67, 68].

SFPs have been described in several insect orders, such as honeybees (Hymenoptera), field crickets (Orthoptera), flies and mosquitoes (Diptera), moths and butterflies (Lepidoptera), and genus *Tribolium* (Coleoptera) [69–71]. To date, no other species of beetles have been analyzed for these proteins as markers for environmental pollution. Thus, *P*. *latreillei* is considered a model organism to evaluate the environmental impacts on the tested SFPs. In this study, a primer set was designed from the sequence of the tiger beetle’s AcPC01 protein available from Genbank [42].

Accessory glands of adult male insects are considered the main organs for producing the non-cellular portion of the sperm [72]. Secretory cells in the accessory gland produce accessory gland proteins (AcPs) that are transmitted to the female with sperms during mating [73]. In our study, a significant downregulation of AcPC01 was observed in males collected from the polluted site. Similarly [74], observed significant inhibition of AcP36DE expression in the accessory glands of male *D*. *melanogaster* treated with organophosphate compounds, dichlorvos and chlorpyrifos. They reported that the chemicals might either inhibit the regulation of AcPs or cause damage to the cells producing them.

The results obtained from MN in the testicular cells of insects collected from the polluted site indicated the intensity of DNA damage. The polluted site possessed significantly higher frequencies of MN than the reference site. The MN illustrate major damage to DNA that cannot be effectively repaired [75, 76]. Klobucar et al. (2003) [77] detected elevated MN frequencies in the hemocytes of caged mussels, *Dreissena polymorpha*, collected from four monitoring sites in river Drava, with different pollution intensities. They reported that MN formation stayed persistent in the cell until the end of its lifespan. Increased numbers of micronuclei indicate a mutagenic and carcinogenic effect in organisms [78, 79]. Offer et al. (2005) [80] stated that the incidence of micronuclei is attributable to the loss of chromosome segments assignable to chromosome breaks or chromosome exchanges. Hence, their formation is a sign of chromosome damage [81, 82]. The incidence of MN could also designate the level of organisms’ sensitivity to toxins [33, 83]. The MN test supported our findings of the nuclear deformities in the ultrastructure observations.

No pathological features were observed in the male reproductive system of *P*. *latreillei*, which represents the same structure as most of the ground beetles among the coleopteran insects. The testes appeared packed with germinal cysts [13, 14, 35].

Our electron micrographs illustrated sperm differentiation, starting from spermatogonia, which exhibited a round nucleus and nucleolus, and a cytoplasm packed with cytoplasmic organelles. Spermatocytes were seen to have a larger nucleus and aggregated mitochondria, preparing for nebenkern formation. There was chromatin condensation in spermatids and dimensioning in the head size through sperm maturation. Similar features were described previously by several researchers [13, 14, 35, 37, 84]. The sperms in *P*. *latreillei* are homologous to tenebrionid sperms. They consist of a conoid acrosome, slender nucleus, centriole, and flagellum with a 9+9+2 pattern. There are two similar mitochondrial derivatives and accessory bodies on each side of the axoneme [13, 35, 85].

At the polluted site, morphological changes in the nucleus and cytoplasm of testicular cells were noticed in most spermatogenic stages. These pathological signs are a consequence of DNA, protein damage, and dysfunction of membranes due to the action of heavy metals [15, 86, 87]. Heavy metals dramatically change the morphology of membranous organelles, such as the mitochondria, endoplasmic reticulum, and plasma and nuclear membranes [87]. Heavy metals sequester in the intracellular compartments of the nuclei and mitochondria, bind membrane and DNA associated proteins, thus altering membrane function as well as DNA repair mechanisms [86]. Metal-induced changes led to cell cycle arrest, cell death, mutation, and alteration in genomic dynamics.

We noticed some degeneration of the flagellar components of spermatids through spermiogenesis, such as axonemal and mitochondrial degeneration. Axonemal degeneration affects sperm motility, which is based on the movement of axonemal microtubules [88]. Mitochondrial degeneration leads to disruption of ATP supply, thus affecting sperm motility [87, 89, 90]. The presence of double tail spermatids was also observed in this study, which may be attributed to the persistence of cytoplasmic bridges that connect the cells throughout spermatogenesis [91]. Agglutinated spermatids and spermatozoa were obvious phenomena recognized in our preparations. This phenomenon results from the coating of antibodies to the sperms, driving them to clump together, and consequently reducing their motility [92].

The presence of dense vesicles and vacuoles in the cytoplasm was another pathological feature in our electron micrographs. High levels of heavy metals can be sequestered as dense vesicles of the lysosomal system [93]. Also, the continuous release of lysosomal hydrolase may result in vacuolated areas in the cytoplasm [94].

The function of the parietal cells in insects is similar to the function of Sertoli cells in mammals, which are being responsible for the nourishment of sperm and phagocytosis of the residual cytoplasm [35, 95]. Hence, the ultrastructure anomalies which were observed in these cells in the polluted group will affect sperm nourishment and lead to the presence of residual cytoplasm. Due the paucity of existing data, our studies have advanced our understanding of the effect of heavy metals on insect spermatogenesis [14, 35].

Finally, heavy metals sequester in particular compartments, such as the nucleus, mitochondria, and ER, which leads to cellular damage associated with changes in gene expression and DNA damage. Thus, *P*. *latreillei* is a good biomonitoring insect for evaluating heavy metal toxicity.

## 5. Conclusion

Humans benefit from ground beetles because they are active decomposers, recycling and removing feces. They also play a critical role as nutrient recyclers, returning organic matter to the soil via multitrophic interactions. Because they are long-lived and maintain a stable population, they are used in biomonitoring programs to monitor adverse environmental conditions. Genotoxic compounds are found in urban areas featuring industrial activities. Heavy metals are one of the possible genotoxic agents that may induce DNA and protein damage as well as ultrastructure anomalies in testicular cells. These aberrations can affect testicular functions. This study advocates a need for proper measures to be taken to lessen increasing environmental pollution in the urban industrial areas.
